# Enhanced Production of a Novel Cyclic Hexapeptide Antibiotic (NW-G01) by *Streptomyces alboflavus* 313 Using Response Surface Methodology

**DOI:** 10.3390/ijms13045230

**Published:** 2012-04-24

**Authors:** Zhengyan Guo, Ling Shen, Zhiqin Ji, Wenjun Wu

**Affiliations:** 1 State Key Laboratory of Crop Stress Biology in Arid Areas, Northwest A & F University, Yangling 712100, China; E-Mail: jizhiqin@nwsuaf.edu.cn; 2 Anhui provincial laboratory of Agro-Food safety, Resources & Environment College, Anhui Agricultural University, Hefei 230036, China; 3 School of Life Sciences, Anhui Agricultural University, Hefei 230036, China; E-Mail: shenling@ahau.edu.cn; 4 Shaanxi Province Key Laboratory Research & Development on Botanical Pesticide, Northwest A & F University, Yangling 712100, China; 5 Key Laboratory of Plant Protection Resources and Pest Integrated Management, Ministry of Education, College of Plant Protection, Northwest A & F University, Yangling 712100, China

**Keywords:** NW-G01, medium optimization, *Streptomyces alboflavus* 313, Plackett-Burman design, response surface methodology

## Abstract

NW-G01, produced by *Streptomyces alboflavus* 313, is a novel cyclic hexapeptide antibiotic with many potential applications, including antimicrobial activity and antitumor agents. This study developed a system for optimizing medium components in order to enhance NW-G01 production. In this study, Plackett-Burman design (PBD) was used to find the key ingredients of medium components, and then response surface methodology (RSM) was implemented to determine their optimal concentrations. The results of PBD revealed that the crucial ingredients related to the production of NW-G01 were (NH_4_)_2_SO_4_, peptone and CaCO_3_. A prediction model has been built in the experiments of central composite design and response surface methodology, and its validation has been further verified. The optimal medium composition was determined (g/L): corn starch 15, glucose 15, peptone 3.80, (NH_4_)_2_SO_4_ 0.06, NaCl 1.5, CaCO_3_ 1.30, MgSO_4_·7H_2_O 0.015, K_2_HPO_4_·3H_2_O 0.015, MnCl_2_·4H_2_O 0.015, FeSO_4_·7H_2_O 0.015, and ZnSO_4_·7H_2_O 0.015. Compared with NW-G01 production (5.707 mg/L) in non-optimized fermentation medium, the production of NW-G01 (15.564 mg/L) in optimized fermentation medium had a 2.73-fold increase.

## 1. Introduction

**Figure 1:**
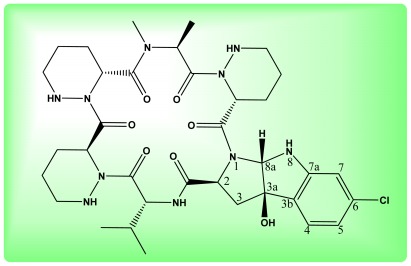
The molecular structure of Novel Cyclic Hexapeptide Antibiotic (NW-G01).

Agricultural antibiotic produced by different species of actinomyces is a biological product from a natural resource. Agricultural antibiotics have been attracting growing interest with the development of environmentally friendly and safe integrated crop management. In our ongoing screening for new bioactive microbial compounds, a novel hexapeptide antibiotic NW-G01 (Figure 1) was obtained by *Streptomyces alboflavus* 313 which is isolated from a soil sample collected from Shaanxi province of China. Absolute structure was determined using a combination of single crystal X-ray diffraction and Marfey’s method finally. The antibiotic incorporated one molecule of valine, *N*-methyl-alanine, chlorinated pyrroloindoline derivative, and three molecules of piperazic acids [1,2]. Interestingly, NW-G01 exhibited strong antibacterial activity against several species of gram-positive bacteria, such as *Bacillus subtilis*, *Bacillus cereus*, and *Staphyloccocus aureus* [3]. In addition, it had strong *in vitro* fungistatic activity against some plant pathogen, such as *Sclerotinia sclerotiorum*, *Botrytis cinerea*, *Coniothyrium diplodiella* and *Exserohlum turcium* [4]. These consequences imply that NW-G01 has the potential to be developed into anti-infection agents or agro-fungicides.

An appropriate fermentation medium is one of the crucial factors in the antibiotic industry, because the medium composition could significantly affect secondary metabolic yield from microorganisms [5]. The conventional method for medium optimization is the “one-factor-at-a-time” approach, which is time consuming, labor expensive, and does not take into consideration the interactions between multiple factors involved when a large number of variables have to be investigated. The main advantage of optimizing the parameters by statistical experimental design is to eliminate these limitations of single factor optimization process [6]. Plackett–Burman is widely used in the statistical designs for the selection of the medium components, which can screen the important variables as well as their significance levels [7]. Following this, factorial design and response surface analysis are used to determine the optimum values of the factors studied based on the initial screening. In the present biotechnology study, several researchers have applied these techniques for the optimization of culture conditions, determination of optimal values of processing parameters and feeding rates [8–11]. Furthermore, these techniques also have been successfully applied to the optimization of medium components and cultivation conditions for metabolic production by *Streptomyces* [12–17] and other microorganisms [18–21]. Response Surface Methodology (RSM) has eased process development and has been of significant use at industrial level, among which central composite design methodology considers the interaction effects among the variables [22].

To the best of our knowledge, the medium requirements for *S. alboflavus* 313 in liquid fermentation have not been demonstrated. Moreover, it is necessary and important to decrease production cost and to enhance productivity. This paper explores the feasibility of production of NW-G01 in liquid fermentation by *S. alboflavus* 313 and its optimization by RSM. Initially, effects of various carbon sources, nitrogen sources and inorganic salts were investigated by a Plackett-Burman design, and subsequently by RSM. Finally, the production of NW-G01 was validated using the optimized condition.

## 2. Results and Discussion

### 2.1. Optimization by Plackett-Burman Design (PBD)

The importance of the eleven components, corn starch, glucose, (NH_4_)_2_SO_4_, peptone, NaCl, CaCO_3_, MgSO_4_·7H_2_O, K_2_HPO_4_·3H_2_O, MnCl_2_·4H_2_O, FeSO_4_·7H_2_O and ZnSO_4_·7H_2_O for NW-G01 production was investigated by PBD. The results showed the effects of these components on the response and significant levels in Tables 1 and 2.

According to statistical analysis of the data by Design expert software, the results showed that only (NH_4_)_2_SO_4_, peptone and CaCO_3_ had confidence levels above 95% (*p* < 0.05) and were considered to influence NW-G01 production significantly. The others had confidence levels below 95% and hence were considered insignificant. In these results, *R*^2^ = 0.9285 indicated that 92.85% of the variability in the response could be explained in the model.

PBD results indicated that the effect of (NH_4_)_2_SO_4_, peptone and CaCO_3_ were negative. Decreasing the three components concentration might result in higher production of antibiotic NW-G01. Thus, the three variables (NH_4_)_2_SO_4_ (*x*_3_), peptone (*x*_4_) and CaCO_3_ (*x*_6_) were selected and their optimal levels were identified further using response surface methodology.

### 2.2. Optimization by Response Surface Methodology

RSM using central composite design (CCD) was applied to determine the optimal levels of the three selected variables that affected the production of NW-G01. The respective low and high levels (g/L) with the coded levels for the factors are defined in Table 3.

The concentrations of the other factors were fixed at zero level as shown in Table 1. Experimental design and results are shown in Table 4.

The experimental results were fitted with the second-order polynomial (Equation (1)):

(1) $$\begin{array}{l} Y=13.57-3.46x_{3}-0.17x_{4}-2.77x_{6}-0.75x_{3}x_{4}-\\ 0.51x_{3}x_{6}+1.09x_{4}x_{6}-1.32{x_{3}}^{2}-1.18{x_{4}}^{2}+0.70{x_{6}}^{2} \end{array}$$

where *Y* was the predicted response, *x*_3_, *x*_4_ and *x*_6_ were coded values of (NH_4_)_2_SO_4_, peptone and CaCO_3_ concentration, respectively.

The statistical significance of Equation (1) was checked by *F*-test, and the ANOVA for response surface quadratic model is summarized in Table 5. The model *F*-value of 222.43 implied the model was significant, the *P*-value was also very low (<0.0001) indicating that there was only a 0.01% chance that a “Model *F*-Value” this large could occur due to noise. The success of the model could be checked by the determination coefficient *R*^2^, which was calculated to be 0.9850, indicating that 98.50% of the variability in the response could be explained by the model. Normally, a regression model, having an *R*^2^-value higher than 0.9, was considered as a high correlation [23]. The present *R*^2^-value, therefore, reflected a very good fit between the observed and predicted responses, and it was considered reasonable to use the regression model to analyze trends of the responses. A lower value of coefficient variation (CV = 3.33%) showed the experiments conducted were precise and reliable [24]. The Lack of fit *P*-value of 0.1095 implied the “Lack of Fit” is not significant relative to the pure error and no-significant lack of fit indicated the model is good.

The significance of the regression coefficients was tested by a *t*-test. The regression coeffiecients and corresponding *p*-values for the model presented in Table 6. Values of “Prob > *F*” less than 0.05 indicate that model terms are significant while values greater than 0.1 indicate that the model terms are not significant [25]. Therefore, among the model terms in the present study, (NH_4_)_2_SO_4_ (*x*_3_) and CaCO_3_ (*x_6_*) were very significant with probability of over 99% while peptone (*x*_4_) was not significant with a probability of over 84%. In Table 6, the results also indicated that the mutual interaction between *x*_3_ and *x*_3_, *x*_4_ and *x*_4_, *x*_6_ and *x*_6_, *x*_3_ and *x*_4_, *x*_3_ and *x*_6_, *x*_4_ and *x*_6_, had a very significant influence on antibiotics NW-G01 production.

The final results showed that among the independent factors, *x*_3_((NH_4_)_2_SO_4_) and *x*_6_(CaCO_3_) had significant effects on antibiotic NW-G01 production and the negative coefficient of them showed a linear effect to decrease antibiotics NW-G01 production. The quadratic term of the three factors and the interaction between *x*_3_((NH_4_)_2_SO_4_), *x*_4_(peptone) and *x*_6_(CaCO_3_) also had a significant effect.

**Figure 2:**
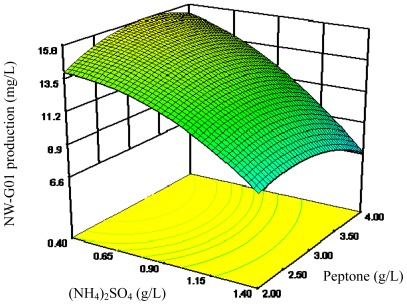
Response surface curve for NW-G01 production by *Streptomyces alboflavus* 313 as a function of (NH_4_)_2_SO_4_ and peptone concentrations, when CaCO_3_ concentration was maintained at 1.00 g/L.

**Figure 3:**
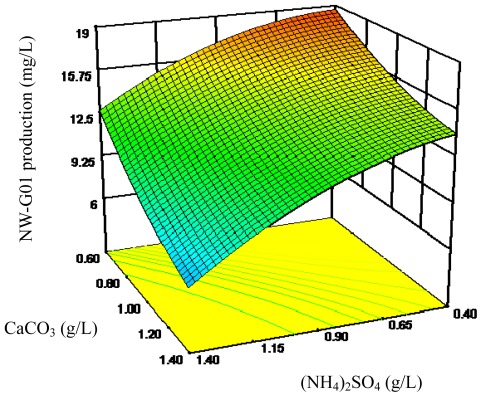
Response surface curve for NW-G01 production by *Streptomyces alboflavus* 313 as a function of (NH_4_)_2_SO_4_ and CaCO_3_ concentrations, when peptone concentration was maintained at 3.00 g/L.

**Figure 4:**
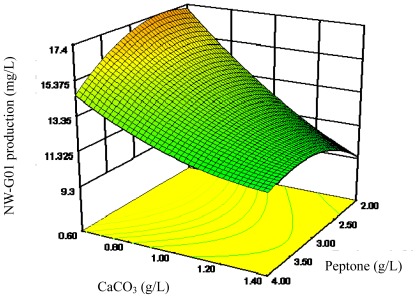
Response surface curve for NW-G01 production by *Streptomyces alboflavus* 313 as a function of peptone and CaCO_3_ concentrations, when (NH_4_)_2_SO_4_ concentration was maintained at 0.90 g/L.

The 3D response surface curves were then plotted to explain the interactions of medium components and the optimum concentration of each component required for the NW-G01 production (Figures 2–4). Each figure presents the effect of two factors while the other factor was held at zero level. These 3D plots and their respective contour plots provided a visual interpretation of the interaction between two factors and facilitate the location of optimum experimental conditions.

### 2.3. Validation of the Optimized Condition

On the basis of medium optimization, the quadratic model predicted that the maximum production of NW-G01 was 15.387 mg/L, when the model predicted the optimal values of test factors in the coded units were *x*_3_ = −1.68, *x*_4_ = 0.80 and *x*_6_ = 0.74, which were 0.06 g/L (NH_4_)_2_SO_4_, 3.80 g/L peptone and 1.30 g/L CaCO_3_, respectively. To verify the predicted results, validation experiments in shake flasks were performed in triplicate testes. Under the optimized medium, the observed experimental value of average NW-G01 concentration was 15.564 mg/L, suggesting that experimental and predicted values (15.387 mg/L) of NW-G01 yield were in good agreement. The concentration was 5.707 mg/L in non-optimized medium, 2.73-fold increase had been obtained, while the growth of the strain in the two media was comparable. This result therefore corroborated the predicted values and the effectiveness of the model, indicating that the optimized medium favors the production of NW-G01.

## 3. Experimental Section

### 3.1. Microorganism

The strain 313 was isolated from the soil samples from the northeast of China, which was identified as *S. alboflavus* 313 based on morphological, physiological characteristics and analysis of the 16S rDNA sequence. The culture was maintained at 4 °C on modified Humic acid-Vitamins (HV) agar slants. The strain was stored in glycerol suspension (30%, v/v) at −20 °C.

### 3.2. Medium and Culture Conditions

Fermentation was performed in two stages: seed growth and antibiotics NW-G01 production. For the seed growth stage, medium from a plate culture was inoculated into 100 mL of seed medium (glucose 20 g/L, peptone 6 g/L, NaCl 2.5 g/L, CaCO_3_ 1 g/L. pH 7.0) in a 500-mL Erlenmeyer flask and grown at 28 °C with 180 rpm on a rotary shaker (ShangHai Fuma Test Equipment Co., Ltd.) for 16 h. Then, 10% (v/v) seed cultures were inoculated into 50-mL production medium in a 250-mL Erlenmeyer flask. The strain was incubated at 28 °C with 180 rpm on a rotary shaker for 108 h. Triplicate experiments were carried out and the mean value was calculated.

In our preliminary experiments, various carbon and nitrogen sources, and inorganic salts were evaluated for the suitability to sustain good NW-G01 production by *S. alboflavus* 313. The results revealed that the major variable affecting the performance of the medium in terms of NW-G01 yield were corn starch, glucose, peptone, (NH_4_)_2_SO_4_, NaCl, CaCO_3_, MgSO_4_·7H_2_O, K_2_HPO_4_·3H_2_O, MnCl_2_·4H_2_O, FeSO_4_·7H_2_O, and ZnSO_4_·7H_2_O. Components were chosen for further optimization. The amount of every component was changed in different experimental processes and the pH of production medium was 7.0.

### 3.3. Analytical Method

After centrifuging the fermentation broth (3000 rpm, 10 min), 1 mL supernatants were filtered (0.45 μm) and analyzed by high performance liquid chromatography (HPLC, Shimadzu 6AD, Kyoto, Japan) using a Sinochrom ODS-BP (5 μm, 4.6 mm × 250 mm) reverse phase column, methanol-water (75/25, v/v) as the mobile phase, flow rate of 1.0 mL/min, monitored by UV detector at 210 nm [1].

### 3.4. Experimental Design and Data Analysis

#### 3.4.1. Plackett-Burman Design (PBD)

PBD was employed for screening the most significant fermentation parameters affecting NW-G01 production with *S. alboflavus* 313. Each independent variable was tested at high and low levels, which are denoted by (+) and (−), respectively. The experimental design with the name, symbol code, and actual levels of the variables is shown in Table 1, whereas Table 2 shows the detail of the design.

#### 3.4.2. Central Composite Design (CCD) and Response Surface Methodology (RSM)

Central composite design (CCD) and response surface methodology (RSM) were employed to optimize the three most significant factors ((NH_4_)_2_SO_4_, peptone, CaCO_3_) for enhancing NW-G01 production. The three independent variables were studied at five different levels (−1.682, −1, 0, 1, 1.682) (Table 3) and a set of 20 experiments were carried out (Table 4).

The factors were coded according to the following equation:

(2) $$x_{i}=\frac{\sum \left(X_{i}-X_{0}\right)}{\Delta X},\;i=1,2,3,\ldots ,k$$

where *x_i_* was the coded independent factor, *X_i_* was the real independent factor, *X_0_* was the value of *X_i_* at the center point and *ΔX* was the step change value.

The response variable (antibiotic production) was explained by the following second-order polynomial equation:

(3) $$Y=\beta_{0}+\sum \beta_{i}x_{i}+\beta_{ii}x_{i}^{2}+\beta_{ij}x_{i}x_{j},\;i=1,2,3,\ldots ,k$$

where *Y* was the predicted response, *β*_0_ was the intercept, *x*_i_ and *x*_j_ were the coded independent factors, *β*_i_ was the linear coefficient, *β*_ii_ was the quadratic coefficient and *β*_ij_ was the interaction coefficient.

#### 3.4.3. Statistical Analysis

Design Expert Version 7.1 (Stat-Ease Inc.: Minneapolis, MN, USA, 2007) was used for the experimental designs and regression analysis of the experimental data. Statistical analysis of the model was performed to evaluate the analysis of variance (ANOVA). The quality of the polynomial model equation was judged statistically by the coefficient of determination *R*^2^, and its statistical significance was determined by an *F*-test. The significance of the regression coefficients was tested by a *t*-test.

#### 3.4.4. Experimental Validation of the Optimized Medium

In order to validate the optimization of medium composition, three tests were carried out using the optimized condition, to confirm the result from the analysis of the response surface.

## 4. Conclusion

Plackett–Burman design and response surface methodology had been proved to be effective on optimization for enhancing NW-G01 production with *S. alboflavus* 313. The final medium composition optimized was (g/L): corn starch 15, glucose 15, peptone 3.80, (NH_4_)_2_SO_4_ 0.06, NaCl 1.5, CaCO_3_ 1.30, MgSO_4_·7H_2_O 0.015, K_2_HPO_4_·3H_2_O 0.015, MnCl_2_·4H_2_O 0.015, FeSO_4_·7H_2_O 0.015, and ZnSO_4_·7H_2_O 0.015, which resulted in an overall 2.73-fold increase compared with that using the non-optimized medium. Validation experiments were also carried out to verify the adequacy and the accuracy of the model, and the results showed that the predicted value agreed with the experimental values well. The optimum culture medium obtained in this experiment laid a foundation for further study with large scale batch fermentation in a fermenter for NW-G01 production from *S. alboflavus* 313.

## Figures and Tables

**Table 1:** The Plackett-Burman design for screening variables in NW-G01 production.

Factors(g/L)	Code	Low	High	Effect	Coefficient	NW-G01 Production	
		Level (−)	Level (+)			*t-*Value	*P*-Value
Intercept					3.18	22.73	0.0004 a
Corn starch	*x*_1_	10	20	0.41	0.2	1.37	0.3046
Glucose	*x*_2_	10	20	0.84	0.42	1.90	0.1163
(NH_4_)_2_SO_4_	*x*_3_	2	4	−4.83	−2.41	65.93	<0.0001^a^
Peptone	*x*_4_	3	6	−1.67	−0.83	7.89	0.0262 a
NaCl	*x*_5_	1	2	0.28	0.14	0.15	0.1000
CaCO_3_	*x*_6_	1	2	−2.15	−1.07	13.08	0.0085 a
MgSO_4_·7H_2_O	*x*_7_	0.01	0.02	0.57	0.29	1.40	0.2341
K_2_HPO_4_·3H_2_O	*x*_8_	0.01	0.02	1.19	0.59	4..01	0.0854
MnCl_2_·4H_2_O	*x*_9_	0.01	0.02	−0.31	−0.16	−1.12	0.4643
FeSO_4_·7H_2_O	*x*_10_	0.01	0.02	−0.88	−0.44	−1.65	0.1493
ZnSO_4_·7H_2_O	*x*_11_	0.01	0.02	−0.57	−0.28	−1.68	0.1919

*R*^2^ = 92.85%, *R*^2^_adj_ = 88.77%;

a Statistically significant at 95% of confidence level.

**Table 2:** The Plackett-Burman design variables (in coded levels) with NW-G01 production as response.

Run	Variable Level											NW-G01 (mg/L)

	*x*_1_	*x*_2_	*x*_3_	*x*_4_	*x*_5_	*x*_6_	*x*_7_	*x*_8_	*x*_9_	*x*_10_	*x*_11_	
1	−1	1	−1	1	1	−1	1	1	1	−1	−1	7.64
2	−1	−1	−1	1	−1	1	1	−1	1	1	1	1.73
3	−1	1	1	−1	1	1	1	−1	−1	−1	1	0.89
4	1	1	−1	−1	−1	1	−1	1	1	−1	1	6.15
5	1	−1	1	1	1	−1	−1	−1	1	−1	1	0.05
6	−1	−1	1	−1	1	1	−1	1	1	1	−1	0.04
7	1	1	−1	1	1	1	−1	−1	−1	1	−1	3.57
8	1	−1	1	1	−1	1	1	1	−1	−1	−1	0.26
9	−1	1	1	1	−1	−1	−1	1	−1	1	1	0.83
10	1	1	1	−1	−1	−1	1	−1	1	1	−1	2.54
11	−1	−1	−1	−1	−1	−1	−1	−1	−1	−1	−1	6.74
12	1	−1	−1	−1	1	−1	1	1	−1	1	1	7.74

**Table 3:** Levels of the factors tested in the central composite design (CCD).

Variables	Units	Symbol Code	Level				

			−1.682	−1	0	1	1.682
(NH_4_)_2_SO_4_	g/L	*x*_3_	0.06	0.4	0.9	1.4	1.74
Peptone	g/L	*x*_4_	1.32	2	3	4	4.68
CaCO_3_	g/L	*x*_6_	0.33	0.6	1	1.4	1.67

**Table 4:** Central composite design matrix for the experimental design and predicted responses for NW-G01 production.

Run	Coded Level			NW-G01 Production(mg/L)	

	*x*_3_	*x*_4_	*x*_6_	Observed	Predicted
1	0	0	0	13.33	13.57
2	−1	−1	1	11.61	11.29
3	1.682	0	0	3.54	4.02
4	0	−1.682	0	10.15	10.50
5	0	0	−1.682	19.94	20.22
6	0	0	0	13.25	13.57
7	−1.682	0	0	15.46	15.65
8	0	0	0	13.47	13.57
9	0	0	0	13.72	13.57
10	1	−1	1	5.07	4.85
11	0	0	0	13.96	13.57
12	1	1	−1	9.74	9.58
13	−1	1	−1	17.22	14.64
14	−1	1	1	14.72	5.19
15	1	1	1	5.62	13.60
16	1	−1	−1	13.99	16.97
17	0	1.682	0	9.62	9.93
18	0	0	1.682	10.49	10.89
19	−1	−1	−1	18.04	17.99
20	0	0	0	13.80	13.57

**Table 5:** Analysis of variance (ANOVA) for the second-order polynomial model.

Source	SS	DF	MS	*F*-Value	Prob > *F*
Model	338.6167	9	37.6241	222.43	<0.0001
Residual	1.6915	10	0.1692		
Lack of Fit	1.2958	5	0.2592	3.27	0.1095
Pure Error	0.3957	5	0.0791		
Cor Total	340.3083	19			

SS, sum of squares; DF, Degree of freedom; MS, mean square. *R*^2^ = 0.9850, *R*^2^_adj_ = 0.9706, *R*^2^_pred_ = 0.9591, CV = 3.33%, PRESS = 10.51.

**Table 6:** Regression results of the central composite design.

Factor	Coefficient	*P*-Value
Intercept	13.57	
*x*_3_	−3.46	<0.0001 a
*x*_4_	−0.17	0.1617
*x*_6_	−2.77	<0.0001 a
*x*_3_*x*_4_	−0.75	0.0004 a
*x*_3_*x*_6_	−0.51	0.0055 a
*x*_4_*x*_6_	1.09	<0.0001 a
*x*_3_^2^	−1.32	<0.0001 a
*x*_4_^2^	−1.18	<0.0001 a
*x*_6_^2^	0.70	<0.0001 a

a Statistically significant at 95% of confidence level.
